# Iridectomy in Glaucoma

**Published:** 1865-06

**Authors:** 


### IRIDECTOMY IN GLAUCOMA.

Prof. Quaglino, of Pavia, at the end of an excellent account of our present knowledge concerning glaucoma, expresses the following opinions upon its curative treatment by iridectomy: 1. Glaucoma, arthritic amaurosis, and arthritic ophthalmia of the older ophthalmologists, are dependent upon one and the same identical morbid process, which only varies by the length or acuteness of its course. 1. The pathological condition which induces chronic and acute glaucoma is choroiditis, with increased secretion of the vitreous humor, and consequent distension of the retina and papilla of the optic nerve, associated with an extraordinary rigidity and hardness of the sclerotica, proper to the senile condition, or induced by an atheromatous and arthritic process at a less advanced age. 3. In acute glaucoma not only is the choroid implicated by the morbid process, but this also extends to the retina, the hyaloid, and the internal membranes, while in chronic glaucoma the choroid is alone in question. 4. The functional phenomena which precede and accompany the development and course of glaucomatous amaurosis are a consequence of the compression which the nervous elements of the retina and the optic nerve undergo, and of their progressive atrophic degeneration. 5. The most prompt and certain means which art possesses for arresting the progress of this disease, and restoring the equilibrium in the pressure of the vitreous humor and the lateral pressure of the vessels of the retina, is iridectomy, the excision of an extensive portion of the iris. 6. Iridectomy may be resorted to with advantage even in cases in which there are evident physical signs of atrophy of the papillæ with excavation, lateral limitation of the field of vision or amblyopia, providing there exists extraordinary hardness of the globe of the eye. In such cases iridectomy at least removes one of the morbid elements (internal pressure) which favors atrophy of the papillæ, and thus frequently arrests the amaurosis. 7. Iridectomy possesses no advantage in very inveterate glaucoma, when the papilla and the vessels have been for a long time atrophied; in cases in which an optic neuritis inducing atrophy of the papillæ has preceded the glaucoma, or when glaucoma is complicated with serious affections of the cerebral optic centres. 8. Iridectomy is of service in cases of obstinate ciliary neuralgia, even when amaurosis has become complete, providing that it depends solely upon compression of the ciliary nerves.—*Brit. and For. Med. Chir. Rev.*, Jan. 1865, from *Annali di Med.*, Oct.—*American Journal of Medical Sciences.*

